# Factors influencing measurement for improvement skills in healthcare staff: trainee, and trainer perspectives

**DOI:** 10.1186/s12909-022-03282-1

**Published:** 2022-04-02

**Authors:** Zuneera Khurshid, Aoife De Brún, Eilish McAuliffe

**Affiliations:** School of Nursing, Midwifery and Health Systems, UCD Centre for Interdisciplinary Research, Education, and Innovation in Health Systems (UCD IRIS), University College Dublin, Dublin, Ireland

## Abstract

**Background:**

Measurement for improvement is an integral component of quality improvement (QI) trainings and demonstrates whether a change resulted in an improvement. Despite its critical role, the development of measurement for improvement skills for QI is relatively under-explored.

**Purpose:**

To explore the training, curricular and contextual factors that influence the development of measurement for improvement skills in healthcare professionals.

**Methods:**

This is a retrospective, qualitative, multiple case study design, based on two QI collaboratives. Trainees and trainers from these programmes participated in semi-structured interviews. A framework drawing on the Kirkpatrick’s evaluation model and the Model for Understanding Success in Quality (MUSIQ) model was developed. The interviews were analysed based on a three-step qualitative thematic analysis method.

**Results:**

A total of 21 participants were interviewed (15 trainees and 6 trainers). Six themes emerged in the analysis of trainee interviewees: impact of differences in job role and hierarchical levels, narrow conception of QI, knowledge disparity between trained and untrained staff, balancing the benefits and burdens of measurement, early adopters of QI driving change and supportive and engaged leadership. Themes in trainer perspectives were knowledge and understanding of measurement, application of PDSA approach to programme design, balancing consistency with adaptation to context, and attributes of sites receptive to change as predictors of development of measurement for improvement skills in staff.

**Conclusion:**

Training alone does not determine the development, sustainability and spread of measurement and QI skills. Instead, it is influenced by a combination of curricular, training, and contextual support structures. Training programmes should be aware of the impact of job role and hierarchy, increased knowledge disparity between trained and untrained staff and trainees equating QI to bundle implementation while designing programmes. Similarly, organisational support through leaders, encouraging staff who have an interest in measurement and a culture receptive to QI also supports development of measurement skills. The study highlights the need for trainees, trainers, and organisations to work together in balancing the benefits and burdens of measurement, leading to sustainable skill development in line with international best practices.

## Introduction

### Problem formulation

Quality improvement (QI) methods provide a systematic approach to health systems for improving the quality of care using iterative change, testing and measurement to demonstrate improvement [1]. Measurement for improvement is one of the basic building blocks of QI and involves conducting repeated tests of change and refining the interventions based on data collection and analysis [2]. Measurement for improvement demonstrates results for the changes being tested so that the interventions can be refined over time [3]. Considering its importance, healthcare organisations dedicate resources to train staff to develop their competencies in implementing QI, measuring, managing, leading, and sustaining change [4].

The role of measurement is one of the key elements in understanding and improving the quality of care [5]. There is also an increasing focus on making the measurement of quality a core part of professional activities for healthcare staff [6]. However, there is limited research specifically investigating the development of measurement for improvement skills in healthcare staff. There are several gaps in research in relation to education and training in QI and measurement for healthcare staff along with the application of learning into practice by healthcare professionals. While QI programmes may lead to improvement in learner knowledge, the impact of QI training programmes on clinical outcomes has not yet been established [7]. The lack of conclusive evidence around success of QI initiatives is also attributed to the poor understanding of contextual factors in research as sometimes successful QI initiatives may fail to transfer to other settings, owing to contextual barriers [8].

Quality measurement is frequently treated as an ancillary matter in healthcare systems’ approach to QI and is often included as an additional check [9]. This lack of interest in measurement requires further exploration to understand the reasons, and to develop evidence-based strategies to promote measurement skills at individual, organisational and health system levels. A systematic review of determinants of developing measurement skills in healthcare staff highlighted that it requires a collective effort from trainers, trainees, the organisations in which the interventions are implemented [10]. Although training and curriculum are important, measurement skill development in staff is influenced by other factors such as staff engagement, strategic approach to QI, organisational support, intervention design, communication, accountability, leadership support and learning networks [10]. This indicates the need to explore the training, curricular and contextual factors in developing staff measurement skills.

### Research aim

There is a need for research to understand the factors that influence the development of measurement for improvement skills in healthcare staff so they can become quality improvers. This research aims to address this gap by exploring the training, curricular and contextual factors that impact the success of measurement for improvement training for healthcare staff based on the perspective of trainees and trainers.

## Methods

### Research approach and reflexivity

This is a retrospective qualitative research based on a multiple-case design. The study is positioned as a constructivist-pragmatist research paradigm as it attempts to make sense of the problem by understanding the context, views of different stakeholders and investigates what works and why. The constructivist view states that reality of a phenomenon is socially constructed, and individuals understand the world in which they live and work by assigning subjective meanings to their experiences and the researcher relies on participant’s views about the phenomenon being studied [11]. Pragmatism believes there are multiple forms of reality and is concerned with the investigation of what works, and then developing solutions based on this knowledge [12]. The research question therefore presents characteristics of both pragmatism and constructivism. The researcher worked closely with the National QI team which included some of the study participants as well. To counter this risk, the researcher employed various reflexive techniques such as maintaining a field journal, questioning assumptions and findings, not involving the national team in the data analysis, and ensuring that conclusions were drawn from only the data, not from conversations outside of the interviews.

### Context

The National (QI) Team of the National Health Service in Ireland drives the training and capacity building of QI and measurement for improvement skills of healthcare staff. The aim of the National QI Team is to promote continuous and meaningful QI in the health service by developing partnerships, offering QI consultations and trainings, and building QI networks. The team facilitates national QI collaboratives for multidisciplinary teams across the health system, based on international best practices. The team has developed a repository of resources such as checklists, templates for developing charts, measurement plan templates, video resources as well as a measurement for improvement curriculum [13]. The curriculum aims to teach staff to identify improvement opportunities, choose measures, develop a measurement plan, collect, analyse, display and interpret findings and act on those findings [13]. It describes four levels of measurement skills, basic understanding for all healthcare staff, skills for those who are part of improvement teams, improvement team leader skills and improvement advisor skills [13].

The study cases are a Pressure Ulcers to Zero Collaborative (PUTZ) and a Clinical Microsystems (MS) collaborative. These were modelled on the Institute for Healthcare Improvement’s Breakthrough Series collaborative approach, which is a short-term learning system, lasting between 6 to 15 months, where different teams from the health system participate in learning days on a focused topic for improvement and then implement this in their own sites during activity periods [14]. The PUTZ collaborative started in March 2017 and its objective was to reduce the number of ward-acquired pressure ulcers by 50% in the participating teams during the six-month collaborative period and to sustain these results over a twelve-month period. A clinical microsystem is defined as a small group of staff who regularly work together to provide care to a group of patients [15]. When combined with QI methodology, clinical microsystems allow the staff to work on improvement initiatives within their departments and patient populations. The MS collaborative began in February 2017 with the purpose of enabling frontline staff in emergency departments to identify and work on improvement initiatives within their departments. Multidisciplinary teams from 15 acute hospitals/sites participated in the PUTZ collaborative. Emergency department teams from ten hospitals/sites participated in the MS collaborative.

### Sampling strategy

The research used a purposive sampling strategy. The office of the National Director for QI acted as a gate keeper and contacted the Assistant Directors of Nursing of sites that participated in the collaboratives. When the sites agreed to participate, the contact details of staff members were provided to the researcher. Five hospital sites were contacted for PUTZ and MS each. For PUTZ, three hospitals sites agreed to participate (7 participants) while two hospitals sites agreed to participate in MS (8 participants). All 6 trainers who were contacted agreed to participate in the study.

### Ethics

The study was deemed low risk as it fulfilled more than one criterion for low-risk studies defined by our institution’s Human Research Ethics Committee. These included retrospective, anonymised data collection on a non-sensitive topic with non-vulnerable participants which was commissioned by the HSE. The study was therefore granted exemption from full ethical review (LS-E-19–108). This low-risk study review outlined detailed study procedures and consent process.

### Data collection methods

Semi-structured interviews were conducted between November 2019 and January 2021. All interviews were audio recorded, transcribed, and anonymised and informed consent was obtained. Interviews were conducted face to face, over the phone and using an online meeting platform.

### Unit of study

**Fig. 1:**
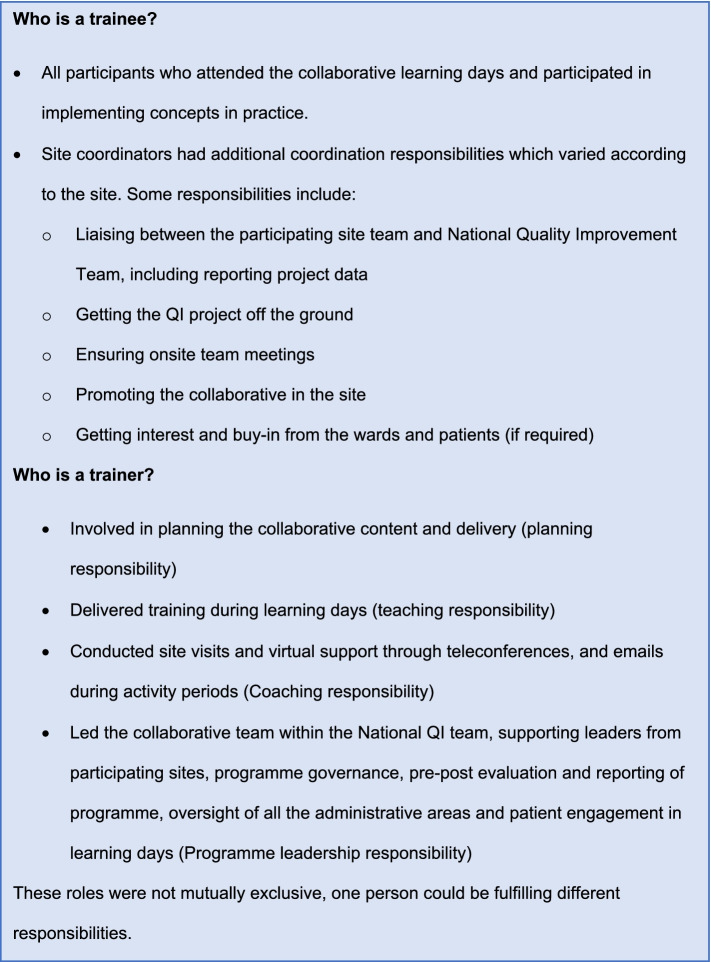
Defining ‘trainer’ and ‘trainee’ roles. A summary of the roles and responsibilities of trainers and trainees as described by the participants

The unit of study is the individual trainers and trainees. A description of roles of trainees and trainers is presented in Fig. 1.

### Data analysis

An evaluation framework was developed for this study based on an adaptation of the Kirkpatrick’s evaluation model [16] and the Model for Understanding Success in Quality (MUSIQ) [17] and this framework informed the development of the interview guide. Further details on this framework are explained in the protocol paper for this work [18]. Data analysis was based on a 3-step thematic coding process. First step was initial open coding, second step was post coding transitions by visually mapping codes and developing categories and third step was another phase of coding to identify dominant codes and emerging themes [19] using NVivo software [20].

### Trustworthiness

To ensure trustworthiness, both trainer and trainee perspectives were included. Lead author conducted the coding, and the emerging codes were discussed with the other two authors in regular sessions and consensus was reached. One trainee from each case and one trainer were contacted via email for member checking (*n* = 3 or 14%) and no additional finding emerged. The study is reported using Standards for Reporting Qualitative Research (SRQR) guidelines [21]. A portion of the data collection was conducted during the COVID-19 pandemic and interview guides were amended to capture participant perceptions about usefulness of the training during COVID-19.

## Results

Fifteen trainees from 5 hospital sites and all 6 trainers involved in the two collaboratives participated in the semi-structured interviews (*n* = 21). The sample included staff from clinical and non-clinical roles including Healthcare Assistants (HCAs), nurses, physiotherapy, and Assistant Directors of Nursing (ADON). Despite the diverse backgrounds of participants, one limitation was that no physician participated in the study and their perspective could not be captured in the study. The characteristics of the sample are summarised in Table 1. The programmes included more participants from the nursing discipline which is also reflected in the sample. Overall, all participants expressed satisfaction with the structure, content, and delivery of the collaboratives. Results of the thematic analysis are organised as trainee perspective and trainer perspective.

**Table 1:** Sample characteristics (*n* = 21)

**Trainee characteristics (*n* = 15)**	
**Programme**	**N**
PUTZ	7
Microsystems	8
**Role in collaborative**	**n**
Site coordinators	3
Trainees	12
**Setting**	**N**
Acute care ward	3
Medical ward	4
Emergency department	8
**Job role (during collaborative)**	**N**
Senior occupational therapist	1
Assistant director of nursing (ADON)	2
Healthcare assistant (HCA)	3
Clinical nurse manager (CNM)	2
Nurse practice development	1
Clinical facilitation	1
Advanced nurse practitioner	2
Staff nurse	2
Senior physiotherapist	1
**Trainer Characteristics (*n* = 6)**	
**Programme**	**N**
Only microsystems	2
Only PUTZ	1
Both	3
**Role in collaborative**	**N**
Programme leads	2
Training/coaching only	4

Summary of characteristics of the trainee (*n* = 15) and trainer (*n* = 6) research participants

### Trainee perspective

The analysis of the trainee interviews revealed six common themes across the two cases: Impact of differences in job role and hierarchical levels, narrow conception of QI, knowledge disparity between trained and untrained staff, balancing the benefits and burdens of measurement, early adopters of QI driving change, and supportive and engaged leadership.

#### Impact of differences in job role and hierarchical levels

Trainee expectations from the programme were influenced by their job role and position in the hierarchy. Frontline staff were interested in benefiting clinically from the programme while those in other roles such as site coordination or practice development, had different learning expectations and goals.

*“There were two different, two different needs, my need necessarily wasn’t the clinical aspect. My need was more how do I, how do motivate the group, how do I get the group”.* (Site Coordinator/ADON: PUTZ)

Those lower in the hierarchy had less access to QI training opportunities which impacted their QI knowledge levels and limited their opportunities to apply learning in future. Sustainability of QI skills was also dependent on whether the job role offered opportunities to apply skills. Participants also perceived a segregation between clinical duties and QI work and believed that managing clinical care is staff’s core duty while QI is peripheral.

*“From the nursing point, on the wards I suppose it’s difficult to get involved with the measurement side of things because you are more into the practical side and the doing”.* (Nurse: PUTZ)

Participants described their peers as only those who were in a similar role to theirs and reported only sharing knowledge with them. The Healthcare Assistants (HCAs) talked about spreading knowledge to other HCAs while nurses focused on reaching out to other nurses. This is indicative of the ingrained, siloed ways of working and could represent an impediment to the spread of QI. The influence of hierarchy sometimes appeared during project group meetings where those lower in the hierarchy felt their opinions could be overlooked. Participants perceived QI to be a specialised role and the responsibility of those much higher in the hierarchy. This common theme was observed across the programmes, teams and organisations participating in the research:

*“Everybody’s idea of QI was that it was done by somebody else in the office, so somebody else a lot higher than all of us”* (Advanced nurse practitioner: MS)

#### Narrow conception of QI

Most participants perceived QI only in terms of bundle implementation, project completion and limiting their use of QI to the tool shown to them during the programme. This was further evidenced by the lack of planning beyond the completion of projects and sustainability was left up to chance:

*“Nah, just fingers crossed and hoped for the best [..] I think they kind of it does sustain itself”* (Clinical facilitator: MS)

The narrow conception of QI was also reflected in participants’ motivation to continue QI efforts associated with the success of the initiatives. This could be challenging as it indicates a restricted understanding of QI methodology, focusing only on positive outcomes and success stories and setting an unrealistic expectation. This was also true for management support, with on-going and future support sometimes contingent on the outcomes of the projects.

There was a perception that QI only helped in certain situations and if staff were frustrated due to infrastructure issues, these bigger issues needed to be fixed before QI can happen. It reflects a perception that QI is a limited value enterprise that can only function when there are no process or system impediments. Another common theme across the programmes, teams and organisations was that participants believed that to maintain skills, it is essential to be a part of formal QI groups and teams as it is not relevant to everyday care delivery.

*“Microsystems is good in the sense that it can give people a chance to maybe join a group when there is a when there is some request of a new group forming”* (Nurse: MS).

The expectation of the programmes was to instil the understanding of QI in trainees so they could spread their knowledge and skills to others and continue doing QI independently. However, participant perceptions did not reflect this broader understanding of QI as many expected to apply their QI skills only when they will be invited to another collaborative-style training in future. This narrow conception of the perceived applicability of QI was also reflected in staff perceptions of rapid changes happening in the health system during COVID-19. Most staff did not believe that QI methodology was appropriate for the pace at which change was happening.

*“Everything was happening so quickly. things were changing by the day you know we never really got time to look at anything in a plan, do, study, act cycle”* (Advanced nurse practitioner: MS)

#### Knowledge disparity between trained and untrained staff

Training participants described a knowledge and skills disparity between those who attended the training and those who did not. The training added to the skills and knowledge of trainees while that of untrained staff was described to remain constant, leading to an increased knowledge gap. This disparity channelled into feelings of inequity in those who did not attend the training.

*“Sometimes people in the other areas felt that they didn’t get the same level of training or education as maybe the people who had been involved in the original project because I suppose they weren’t involved in the study days”* (Site coordinator: PUTZ)

Participants also believed that because of this knowledge disparity, untrained staff could not see the benefit of the projects and were less engaged and motivated.

*“Those of us who attended the meetings (collaborative training days) were much more keen and much more involved and wanting to see the change happen”* (Advanced nurse practitioner: MS)

This led the trainees to believe that everyone should have received the training directly which would have created a greater spread of knowledge rather than them trying to spread it to their colleagues. This perception represents a significant barrier to the programmes’ anticipation of the sharing of knowledge of measurement and QI from trained to untrained staff.

#### Balancing the benefits and burdens of measurement

Whilst participants acknowledged the importance of measurement in achieving clinical and project outcomes, most were not keen on learning and implementing it. The extensive data collection required in the collaboratives was described as a burden in addition to daily duties and competing work demands. It was also noted that staff can lose interest in measurement overtime, which may impact sustainability.

*“I do think it is difficult for staff on the floor just with relation to the run charts and those particular measurements it’s difficult to be able to get the staff on the floor to engage and participate in that part due to the busyness of the work and the environment that we are in the acute hospitals”* (Site coordinator/ADON: PUTZ)

Participants perceived measurement to be a specialised area and did not consider it relevant and beneficial outside their projects which may have also added to the perception of measurement as a burden.

*“You have to be doing active research I think within the department for it to use it (measurement) you know, but we don’t do any research, unless someone was doing research in college or something, but we haven’t done a huge amount of that kind of stuff outside our QI group”* (Clinical facilitator: MS)

Many participants found measurement, especially quantitative measurement, intimidating and assumed it was only intended for advanced learners or those interested in measurement. Due to this, measurement responsibility often was assigned to a team member who had some previous experience or interest in measurement. This led to the emergence of data experts within the team but also increased the risk of other team members not developing a basic understanding of measurement which can be a threat to sustainability.

*“From a mathematical point of view that was [..] beyond the grasp of a lot of the normal folks there to be honest you know, it was, it was, yeah I thought it was too much”* (Advanced nurse practitioner: MS)

#### Early adopters of QI driving change

While some staff members found QI cumbersome, there were a few who were committed to leading change and were the early adopters of QI. The QI early adopters willingly utilised their personal time for project completion; regularly attended meetings, recruited others, and supported implementation.

*“It was the staff’s own time so they would come in on their days off or they would stay longer than their actual shift to collect data to do surveys that kind of thing anything we implemented money wise it may have been bought by the staff themselves”* (clinical facilitator: MS)

With the demanding nature of work for healthcare professionals, not all participants shared the same enthusiasm for implementing change. The QI early adopters demonstrated improvement through their projects which made the leap easier for their colleagues and supported spread.

*“Being able to show the improvement results was probably one of the things that helped the most (for spread) because I suppose they could see that it had an impact for the patients with improved outcomes”* (Site coordinator/Nurse practice developer: PUTZ)

With time, only a core group of staff who were genuinely interested in QI remained engaged with the projects. Early adopters also contributed towards changing negative mind-sets and attitudes about QI/measurement prevalent among staff and constantly reminded staff to adopt and implement the new methods. An additional observation was that those who did not embrace QI seemed to have an external locus of control, attributing their failures and difficulties to external factors while the early adopters of QI took responsibility of their actions and had an internal locus of control.

*“We failed obviously because of the cramped situation, we don’t have any resources and we don’t have any time now that will be available because the training isn’t being run this year. so, if those things aren’t in place, it’s impossible to run these programmes*” (Physiotherapist: MS)

#### Supportive and engaged leadership

Participants described that supportive and engaged leaders played a critical role by providing dedicated time to staff to attend training days and implement QI, encouraging and praising their work and facilitating access to the required resources. Leadership support also played a role in spread by encouraging staff to share their knowledge with others:

*“It’s very good support here in my new job and that my manager in in my supervision session is always pushing me to present more stuff to share knowledge with the rest of the team, to do different projects and to present what works”* (Senior Occupational Therapist: PUTZ)

Presence of engaged leaders who are interested in the work of QI teams and their outcomes also created accountability for the projects. If leaders are passive, QI teams remain unaware of the organisational priorities and resource constraints and focus on projects which are unable to get leadership support later-on, leading to disappointment and wasted time. Support and encouragement of leaders motivated QI teams to complete projects and promoted sustainability:

*“Sometimes you get a bit down when things aren’t going your way so you are like couldn’t be bothered doing this anymore because it’s not really working but then you have our CNM3 and our ADON who are part of this group and they’ll be like oh no come on we will just need to kind of work through and get on with it” (Clinical facilitator: MS)*

Some participants described leadership support as the most important factor without which, any amount of measurement and data collection can lead to change.

*“Even if we found out this is the number of interruptions (handover project), we were getting during our handover period then if the management didn’t support us with our solutions to those problems it wouldn’t have happened”. (Staff nurse: MS)*

### Trainer perspective

Four main themes emerged from trainer interviews. These themes were knowledge and understanding of measurement, application of PDSA approach to programme design, balancing consistency with adaptation to context and attributes of sites more receptive to change.

#### Knowledge and understanding of measurement

An important finding from the interviews was that trainee’s pre-conceptions about measurement impacted their ability to learn. Even though staff spent a considerable time in measurement activities such as recording vital signs and charting, they did not recognise it as measurement. An underlying assumption of the programme and curriculum is that the staff would be aware of basic concepts such as calculating averages or percentages however, the trainers often spent time covering these basics. In the PUTZ programme, staff collected baseline data for a month before attending training sessions and during this time, they developed perceptions about measurement which they brought to the programme. Overcoming negative attitudes and perceptions about measurement therefore was an additional barrier that trainers had to overcome.

*“One of the staff nurses who was quite sceptical about the whole programme initially [..] she thought we were kind of coming into tell her how to do her job [..] and a bit worried about what we were going to be doing with all this data all this data we were collating through the measurement and was it going to be used against them”* (Trainer: PUTZ)

Trainers also perceived that sometimes trainees held defensive and biased views about their data which the trainers had to recognise and overcome.

*“They (trainees) want to use certain parts of the data to reinforce that [..] people tend to dismiss data that doesn’t fit with their worldview in the first place”* (Trainer: PUTZ & MS)

Trainers also noticed that certain staff categories such as consultants were more aware of and interested in measurement. They also stressed that it is not important for all team members to be experts in measurement however the presence of data experts does not absolve other team members from developing a shared understanding of data. From the perspective of programme design, measurement was needed to demonstrate the benefit of the collaborative to the National Health Service and cementing its continuity.

*“I am not so sure if the investment would have continued from collaborative to collaborative to collaborative in the absence of the measures that were provided”* (Programme lead/trainer: PUTZ)

#### Application of PDSA approach to programme design

The iteratively evolving nature of the programmes where the experience of previous trainings informed the content of the following ones like a PDSA cycle was frequently discussed by trainers. The national QI team strives to incorporate and encourage PDSA thinking and application. In the programme design and delivery, the trainers implemented this PDSA mind-set. They used the learning from previous QI collaboratives to inform the PUTZ and MS collaboratives. Based on participant feedback collected through evaluation forms before and after the training sessions, trainer observations and team after action reviews, the trainers were able to improve subsequent training sessions’ content, and delivery. Sometimes additional supports were also provided to participants which were not part of the original programme design, based on trainee feedback*.*

*“There was I suppose previous, probably the PDSA of what worked and didn’t work for phase 1 and phase 2 (for PUTZ) plus there was a lot of engagement with international QI colleagues around resources” (*Trainer: PUTZ*)*

Formal evaluations were conducted regularly to track individual and group skill development over time, something which offered valuable insights for steering the programme. At the end of each session, trainers also evaluated their own performance to evolve and adapt, and they collectively reflected to inform programme structure.

*“I suppose reviewing how the day would have gone what we could have done differently and what we could improve and what we needed to adapt, and we did that as each time that we had a training session”* (Trainer/programme lead: MS)

#### Balancing consistency with adaptation to context

Trainers articulated the importance of delivering a QI and measurement message that was aligned with international best practices but adapted to the national policy context. To ensure this, the microsystems and PUTZ programme leads were trained in best practices from Dartmouth Institute and the Institute of Healthcare Improvement.

*“Using a known improvement, tested methodology and that was the IHI improvement cycle […] the framework for improvement in healthcare to ensure the correct governance, to ensure that we were engaging the people we needed to engage, to ensure we were using the proper methods, to ensure that our approach to measurement was correct”* (Trainer/programme lead: PUTZ)

The programme leads, along with other trainers, adapted these best practices to the needs of the Irish healthcare system and national policy.

*“Coming from the quality improvement team we had the framework then to look at. We had our six drivers [identified in the National QI Framework for QI] and very much then you looked at the programme through the leadership governance and how that was going to be ticked off”* (Trainer/programme lead: MS)

To ensure the delivery of an aligned message, the trainers established operational definitions of measurement on day one to promote consistency. Even though trainers belonged to different specialisms, while delivering the training they worked collectively to deliver a consistent message that would help trainees understand QI and measurement. Trainers perceived a need for more international collaborations that would align the measurement and QI teaching in Ireland with international perspectives.

*“There are people in the NHS improvement and there are people who do use R or so mainly it would be support from like groups likes that or networking or interaction with people like that so we could learn from each other because we do teach similar things”* (Trainer: PUTZ & MS)

The rapid changes happening in the health system during COVID-19 also impacted QI training activities and the trainers were actively considering how the training content, delivery and timing could be adapted to this new context.

#### Attributes of sites more receptive to change

Trainers described attributes of sites that they perceived to be more receptive to change. Having strong leadership was one of these distinguishing factors as formal leaders in more perceptive sites supported collaborative teams, celebrated wins, and maintained accountability. Such sites also had informal leaders driving change as well.

*“Show their (leader) support and not just saying do you want to be a part of it but you know also I think in challenging, so it doesn’t go wrong. High challenge high support using that where necessary and also celebrating small wins and small successes”* (Programme lead: PUTZ)

More receptive sites selected people who were interested in QI and measurement to attend the training, built a stable team, and took ownership of the collaboratives by ensuring governance of the projects and data collected so that initiatives did not fade with time. Trainers also observed that such sites had a person-centric culture.

*“Sites that would have been more successful I suppose had a very well-established team from the outset so they would have spent time thinking about who needed to be on the multidisciplinary team who needed to attend the training and they chose wisely”* (Programme lead: MS)

Measurement also emerged as an important factor as teams and sites that embraced measurement and had a measurement expert were perceived as more perceptive by trainers. This was also indicative of a supportive culture of QI in the organisation where QI teams were supported and purposefully selected and were facilitated to meet regularly which supported the development of measurement skills:

*“There were definitely teams who embraced the measurement component of the training far better than others. Where it worked really well where there was somebody with a particular interest in measurement”* (Trainer: PUTZ & MS)

Comparing the findings across the two cases, PUTZ focused on a common measurable goal of reducing pressure ulcers across participating sites by implementing a bundle, microsystems had a broader focus where participants identified problems from their respective emergency departments and developed instruments such as surveys on their own. On an implementation level, microsystems’ participants worked on a diverse range of projects, but some sites struggled with project selection and ended up selecting large projects that required long-term resource and time commitments which could not be completed successfully and turned into a source of frustration. Due to the diverse nature of projects, and participants, it was more challenging for trainers to develop an appropriate pitch and anticipate trainee queries for microsystems. On the other hand, PUTZ offered a more structured approach in which participants did not have to explore improvement opportunities on their own and allowed participants to have a narrow focus on the bundle increased the risk of participants equating QI to bundle implementation.

## Discussion

This study addressed a gap in research around the factors that influence the development of measurement for improvement skills in healthcare staff. The findings showed that the common premise that staff trained in QI collaboratives will spread their knowledge to others may not hold true owing to the negative influences of hierarchy. Another significant finding was that training attendance may lead to increase in skill gap between those who attended the training and those who did not. The discussion is organised into training, curricular and contextual factors.

### Training factors

Overall, participants expressed satisfaction with the PUTZ and MS programmes. The PUTZ and MS programmes were inherently different in their objectives even though both followed a collaborative style structure. While PUTZ had a focused objective of pressure ulcer reduction, MS required participants to identify areas of improvement and implement change. Previous research suggests that while improving quality through clinical microsystems, sustainability can be challenging and smaller projects with clear parameters tend to be more successful than larger ones [22]. Our results also showed that some teams in MS struggled with selecting appropriate projects. In PUTZ there is a risk of participants focusing too narrowly on project completion. This highlights an important area of consideration for programmes to help participants achieve the balance between being too narrowly focused and becoming overambitious in project selection.

A unique finding of this study was the increased knowledge disparity and feelings of inequity created between those who attend the training and those who were not part of the training. As the programmes improved the knowledge and skills of the trainees, the knowledge and skills gap with colleagues who did not attend the training increased, which created feelings of inequity. Attending the training was viewed as a high-quality learning opportunity while learning from colleagues through spread was not perceived to be of the same value, thus raising questions around the usefulness of a cascade or knowledge sharing approach for spread. Participants in our study believed that QI training is a dose to be administered by the national team repeatedly to retain skills which presents a gap between trainer/programme and trainee expectations.

### Curricular factors

Trainers embodied the national expectation that QI is important for all healthcare staff however trainees entered the programme with variable clinical and QI experiences, job roles and position in the hierarchy which impacted their learning expectations and QI/measurement perceptions and not everyone considered it useful. This was in line with previous evidence that suggests that trainee background such as previous professional training, workplace experiences and opportunities to engage in QI, impacts their learning expectations, interests and needs from the programme [23].

QI is a systematic methodology for making changes; bundle implementation and projects are one of the means of conducting QI. Previous studies have evidenced that improvement work can often be reduced to the use of tools or a limited application of principles [24] and improvement activities remain localised and do not plug into the broader organisational resources and structures [25]. This was also true for this study where many participants described a narrow conception of quality, focusing on bundle implementation and project execution without much attention to underlying QI methodology and the wider relevance of QI.

Whilst participants recognised the importance of measurement in QI, most still felt daunted by it. Previous literature has suggested that even though improving quality is much discussed, there is a reticence towards engaging in measurement because it is time consuming and the process seems intimidating [26]. The participants of this study echoed this perception, finding measurement burdensome and not relevant to their clinical role. Staff in some sites struggled to take ownership of the data once the collaboratives ended, suggesting measurement was only being undertaken for project completion. Trainers hoped that the teams would have a shared understanding of measurement and look at data collectively, yet measurement was still viewed as an additional task and ended up being assigned to a specific team member.

As trainees had differing levels of interest and knowledge, it was challenging for trainers to pitch the content at a suitable level for all those present. Presenting all measurement concepts to the entire audience often left those who did not see its relevance to their roles confused and intimidated. However, trainers also recognised that not everyone needs to learn advanced measurement concepts. This may highlight a need to balance the efforts to provide some level of measurement training to all staff in the health system as outlined in the national QI training curriculum, versus nurturing staff who are interested in learning and using measurement.

### Contextual factors

Literature suggests that promotion of QI among healthcare staff through activities such as QI training does not necessarily result in an improved understanding of QI and these activities may remain limited to experts and early adopters [27]. This was also reflected in the findings of this study. Training and content alone did not predict long term sustainability and spread of measurement skills; it was also influenced by job role and position in hierarchy of trainees, whether trainees developed a broad or narrow understanding of QI, knowledge disparity between trained and untrained staff, how trainees balanced between the benefits and burden of measurement, the role of early adopters of QI and the presence or absence of supportive and engaged leadership. The importance of leadership support was echoed by the trainers, and they perceived the development of measurement skills to be influenced by trainee background knowledge and understanding of measurement, iteratively testing, and refining programmes, adapting best practices to context and receptivity of the sites to change.

Previous studies have suggested that healthcare professionals often artificially differentiate and separate QI from clinical work [28]. This was visible in our study findings as those in frontline roles such as nurses and HCAs were more interested in the clinical benefit of the programme rather than learning QI and are often unable to make the connection between the importance of QI in improving clinical care. This represents a barrier in achieving the health system aim of such programmes to educate all healthcare staff in basic QI and measurement principles. This indicates that weaknesses in measurement skills may be a symptom of deeper, organisational and health system-wide cultural problems. Similarly, previous research studies have demonstrated that there is variation in the ability of learners from different professional backgrounds to conduct projects and influence system level improvements as those in formal QI roles are more involved in QI implementation [29]. This also holds true for the participants in this study. Those in senior/managerial roles had more exposure to and interest in QI while the others focused on clinical roles and believed QI is a specialised job for those higher in the hierarchy.

This study also uncovered another barrier that may hinder the spread and sustainability of QI/measurement. An expectation of the programme was that trainees would share their knowledge with other colleagues however; the interviews revealed that trainees only consider those in similar roles as peers. This highlighted deeper, organisation and health system level issues including siloed ways of working, negative impacts of hierarchies in organisations and communication breakdowns. Literature also suggests that siloed mentality in health services leads to creation of horizontal as well as vertical silos which impact information sharing and adaptation of innovations [30]. This was observed in the trainee interviews as staff only viewed those in similar roles as their peers and mainly shared their knowledge with those whom they considered their peers. This could lead to pockets of improvement within peer groups rather than a multidisciplinary team approach to QI and equal spread of knowledge. QI literature also suggests that planning for quality, sustainability and spread are often neglected, and many initiatives do not spread beyond the original unit [31].

Lucas and Nacer have emphasised the role of improvement habits of learning, influencing, resilience, creativity, and systems thinking have been highlighted as crucial in sustaining improvements [32]. Our study also detected the importance of QI early adopters, individuals who spearhead quality in their respective sites. Additionally, the results of this study indicated a relationship between locus of control and QI where those with an external locus of control are likely to blame failures on external factors. The importance of enthusiastic leadership that allocates time for QI, plans for spread and sustainability and engages staff has been identified as crucial to the success of QI initiatives by previous research studies [33]. In this study, the role of leadership support was recognised by trainees and trainers.

**Fig. 2:**
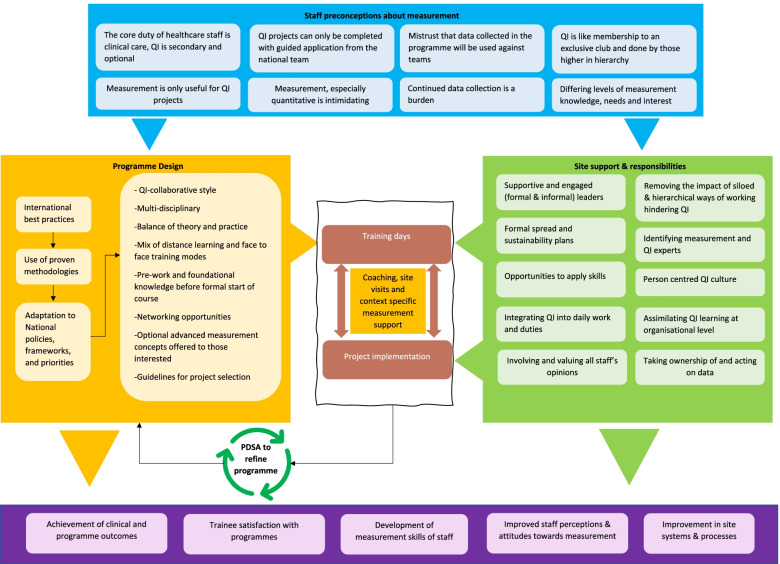
Curricular, training, and contextual factors influencing measurement for improvement skills. A summary of the curricular, training, and contextual factors that impact the development and use of measurement for improvement skills in healthcare staff

Studies conducted during the COVID-19 pandemic have shown that with the onset of the pandemic, healthcare organisations were forced to halt their improvement efforts and all PDSA cycles were disrupted [34]. This was also observed in the participant interviews that were completed during the pandemic. An evidence review exploring virtual adaptation of QI programmes for healthcare staff concluded that virtual training can be an effective alternative to face-to-face QI education and will play a significant role in building QI competency in healthcare staff during and after the pandemic [35]. The participants in this study also believed that virtual QI training can be as effective as face-to-face education and overcome barriers of traditional programmes. Previous studies have confirmed that although technical skills are important for successful QI interventions, the ability of staff to adapt these skills to their context should be of paramount concern [36]. This was also observed in this study as it highlighted that staff measurement for improvement skills may be impacted by a variety of factors apart from training. QI trainings are complex social interventions implemented in a sociotechnical healthcare system and evaluation methods should consider complex qualitative approaches that explore what works, why it works, for whom it works and under what circumstances [37]. This study developed a customised framework that looked beyond the clinical and training aspects and also focused on the complex underlying processes that impact sustainability and spread of QI and measurement for improvement skills. No such study dissecting QI collaboratives to probe factors impacting measurement for improvement has been conducted previously. Rather than only evaluating the human and environmental factors, the study offered an expanded understanding of sociological perspectives to understand the context and incorporated multiple perspectives to provide a layered understanding of measurement and QI which was identified as an area of further research in earlier studies [38]. A summary of the study findings is presented in Fig. 2.

Although this research is based in Ireland, healthcare systems across various countries including United Kingdom, Australia, Canada, United States and New Zealand have developed various resources and toolkits to support the development of measurement for improvement skills in staff [39–42]. This highlights the importance of the global role of measurement for improvement in maintaining a focus on QI and warrants the need for further research. Research suggests that implementing change in healthcare teams is influenced by the existing power structures and ability of leaders to exert their authority to endorse change [43] which requires further exploration in the context of QI implementation. Another area for further research is to explore who healthcare staff consider their peers and why and its impact on the spread of QI/measurement knowledge. There is a need to study the knowledge disparity created between those who were trained and those who were not. This research can also inform policy for curriculum and programme revisions and cataloguing the characteristics of individuals and sites more receptive to QI and measurement. Another critical area highlighted is in relation to staff perception of irrelevance of QI and measurement during COVID-19 when in practice; there have been remarkable stories in the health system about QI in crisis and rapid changes. It will be valuable to explore the factors and mechanisms that motivated and supported staff to engage in rapid improvements during the pandemic, despite their reluctance to engage in QI normally.

### Strengths and limitations

The study has practical and methodological implications in addressing the research gap in exploring factors that influence the development of measurement skills in healthcare staff for QI. The study acknowledges the importance of moving beyond the use of uniform evaluation approaches towards using customised frameworks to understand a broader array of influencing factors. A limitation of the study is that both the programmes were completed more than two years ago making recruitment challenging, resulting in a smaller sample size than desired. This time lag could have led to recall bias. No physicians are part of the sample even though they participated in the collaborative. Physicians’ perspectives may have proved useful in further elaborating the results in relation to perceived segregation between clinical and QI duties.

## Conclusion

The study presents an exploration of trainer and trainee perspectives about developing measurement for improvement skills in healthcare staff. From the perspective of trainees, impact of differences in job title and hierarchical levels, narrow conception of QI, knowledge disparity between trained and untrained staff, balancing the benefits and burden of measurement, early adopters of QI driving change and supportive and engaged leadership emerged as the major factors influencing measurement for improvement skills in healthcare staff across the two programmes. The trainers perceived knowledge and understanding of measurement, application of PDSA approach to programme design, balancing consistency with adaptation to context and QI receptivity of sites as predictors of developing measurement for improvement skills in staff. The study findings highlight that although measurement is a core component of QI, training alone does not determine the sustainability and spread of measurement and QI skills. It is a combination of curricular, training, and contextual factors. Measurement for improvement skills are also influenced by individual-level factors such as interest in measurement, perceptions about measurement and locus of control. The study also highlights the importance of expanding evaluation beyond clinical outcomes and bundle implementation and exploring underlying sociological factors to develop a deeper understanding of QI and measurement.  

## Data Availability

The datasets generated and/or analysed during the current study are not publicly available due participant privacy/consent conditions who provided consent to use their answers only for this research but are available from the corresponding author on reasonable request.

## Abbreviations

QI: Quality Improvement
COVID-19: Coronavirus Disease 2019
PDSA: Plan-Do-Study-Act
HSE: Health Service Executive
